# Prospective double-blind randomized study on the efficacy and safety of an n-3 fatty acid enriched intravenous fat emulsion in postsurgical gastric and colorectal cancer patients

**DOI:** 10.1186/1475-2891-14-9

**Published:** 2015-01-21

**Authors:** Cheng-Jen Ma, Jin-Ming Wu, Hsiang-Lin Tsai, Ching-Wen Huang, Chien-Yu Lu, Li-Chu Sun, Ying-Ling Shih, Chao-Wen Chen, Jui-Fen Chuang, Ming-Hsun Wu, Ming-Yang Wang, Ming-Tsan Lin, Jaw-Yuan Wang

**Affiliations:** Division of Gastroenterology and General Surgery, Department of Surgery, Kaohsiung Medical University Hospital, Kaohsiung Medical University, No. 100 Tzyou 1st Road, Kaohsiung, 807 Taiwan; Institute of Clinical Medicine, Kaohsiung Medical University, Kaohsiung, Taiwan; Nutrition Support Team, Kaohsiung Medical University Hospital, Kaohsiung, Taiwan; Graduate Institute of Clinical Medicine, Kaohsiung Medical University, Kaohsiung, Taiwan; Department of Surgery, National Taiwan University Hospital, National Taiwan University, No. 7 Chung-Shan South Road, Taipei, 100 Taiwan; Division of General Medicine Surgery, Department of Surgery, Kaohsiung Medical University Hospital, Kaohsiung Medical University, Kaohsiung, Taiwan; School of Medical and Health Sciences, Fooyin University, Kaohsiung, Taiwan; Department of Surgery, Kaohsiung Municipal Hsiao-Kang Hospital, Kaohsiung Medical University, Kaohsiung, Taiwan; Division of Gastroenterology, Department of Internal Medicine, Kaohsiung Medical University Hospital, Kaohsiung Medical University, Kaohsiung, Taiwan; Department of Internal Medicine, College of Medicine, Kaohsiung Medical University, Kaohsiung, Taiwan; Department of Nursing, Kaohsiung Medical University Hospital, Kaohsiung Medical University, Kaohsiung, Taiwan; Division of Trauma surgery, Department of Surgery, Kaohsiung Medical University Hospital, Kaohsiung Medical University, Kaohsiung, Taiwan; Department of Emergency Medicine, Kaohsiung Medical University Hospital, Kaohsiung Medical University, Kaohsiung, Taiwan; Department of Pharmacy, Kaohsiung Medical University Hospital, Kaohsiung Medical University, Kaohsiung, Taiwan; Department of Surgery, Faculty of Medicine, College of Medicine, Kaohsiung Medical University, Kaohsiung, Taiwan; Cancer Center, Department of Surgery, Kaohsiung Medical University Hospital, Kaohsiung Medical University, Kaohsiung, Taiwan; Center for Biomarkers and Biotech Drugs, Kaohsiung Medical University, Kaohsiung, Taiwan

**Keywords:** Lipid emulsion, n-3 fatty acids, LCT/MCT, Fish oils, Gastric cancer, Colorectal cancer

## Abstract

**Background:**

A lipid emulsion composed of soybean oil (long-chain triglycerides, LCT), medium-chain triglycerides (MCT) and n-3 poly-unsaturated fatty acids (PUFAs) was evaluated for immune-modulation efficacy, safety, and tolerance in patients undergoing major surgery for gastric and colorectal cancer.

**Methods:**

In a prospective, randomized, double-blind study, 99 patients with gastric and colorectal cancer receiving elective surgery were recruited and randomly assigned to either the study group, receiving the n-3 PUFAs enriched intravenous fat emulsion (IVFE), or the control group, receiving a lipid emulsion comprised of soybean oil and MCTs (0.8 – 1.5 g · kg^-1^ · day^-1^) as part of total parenteral nutrition (TPN) regimen from surgery (day -1) up to post-operative day 7. Safety and efficacy parameters were assessed on day -1 and post-operative visits on day 1, 3, and 7. Adverse events were documented daily and compared between the groups.

**Results:**

Pro-inflammatory markers, laboratory parameters, and adverse events did not differ prominently between the 2 groups, with the exception of net changes (day 7 minus day -1) of free fatty acids (FFAs), triglyceride, and high-density lipoprotein (HDL). Net decrease of FFAs was remarkably higher in the study group, while the net increase of triglyceride and decrease of HDL was significantly lower.

**Conclusions:**

The n-3 PUFA-enriched IVFE showed improvements in lipid metabolism. In respect of efficacy, safety and tolerance both IVFE were comparable. In patients with severe stress, there is an inflammation-attenuating effect of n-3 PUFAs. Further, adequately powered clinical trials will be necessary to address this question in postsurgical GI cancer patients.

**Trial registration:**

US ClinicalTrials.gov NCT00798447.

## Background

Surgical injury is followed by profound changes in endocrine metabolic function and various host defense mechanisms leading to catabolism, immunosuppression, ileus, impaired pulmonary function, and hypoxemia. Nutritional intervention may substantially alter the immune function with potential impact on the post-operative morbidity [1]. Patients with gastrointestinal cancer undergoing major surgery are usually malnourished before surgery and will sustain a worsening of this status because of prohibited enteral nutrition after digestive surgery as well as surgery induced stress metabolism.

Malnutrition due to preoperative poor gastrointestinal function and/or postoperative stress metabolism leads to an increase in morbidity and mortality by mechanisms impairing patients’ immune system. Analyzing the nutritional status adequately allows implementation of appropriate nutrition therapy. The nutritional regimen will usually be composed to provide the basic requirements of patients and thus should contain amino acids, glucose and lipids as well as micronutrients and electrolytes. In addition to their role in providing energy, omega 3 polyunsaturated fatty acids (n-3 PUFAs) have additional features, such as the modulation of the metabolic and inflammatory responses that are of benefit to malnourished patients receiving surgery. N-3 PUFAs are preferentially incorporated into cell membrane phospholipids, influence secondary messenger synthesis and modulate the expression of certain adhesion molecules at the surface of endothelial cells, monocytes and lymphocytes [2, 3]. They have been shown to influence cell membrane fluidity and permeability and to modify the cell membrane receptors and enzymes activity [4, 5]. N-3 PUFAs compete with arachidonic acid to produce prostaglandins, leukotrienes, and thromboxanes with less inflammatory and less thrombotic properties [6–8]. It thus appears that n-3 PUFAs may regulate pro- and anti-inflammatory cytokine gene expression and might be a promising therapy in conditions characterized by inappropriate pro-inflammatory activity [9]. Studies undertaken with n-3 PUFAs containing parenteral lipid emulsions in patients undergoing major abdominal surgery [10, 11], in patients undergoing surgery for aortic aneurism repair [12], and in patients with acute respiratory distress syndrome (ARDS) [13, 14], have proven clinically to reduce length of post-operative hospital stay, frequency of post-operative complications, as well as to lower the levels of pro-inflammatory factors such as leukotriene B4 (LTB4) [14] and Interleukin 6 (IL-6) [15].

The aforementioned data and scientific background suggest that the application of lipid emulsions containing n-3 PUFAs before and after surgery may show beneficial effects. Herein, we had conducted a phase III, prospective, randomized, double-blind, bi-centric, active-controlled, parallel group study to evaluate the efficacy and safety of the n-3 PUFAs containing lipid emulsion in patients undergoing major surgery for gastric and colorectal cancer.

## Methods

The protocol was approved by the local ethics committees ((R9746) IRB-951008) and was carried out in accordance with the Declaration of Helsinki of 1975 as revised in 1996. Written informed consents of patients were obtained before the study was conducted. The study was registered at clinicaltrials.gov under the identification code ID: NCT00798447 and the trial name: Efficacy and Safety of a PN Regimen Containing n-3 Fatty Acid in Patients Considered after GI Surgery.

In this prospective randomized, double-blind study, patients ≧ 18 years of age in Kaohsiung Medical University Hospital (KMUH) and National Taiwan University Hospital (NTUH) following elective radical surgery for gastric and colorectal cancer in open method from January 2009 to October 2010 were screened for eligibility in our study. The following patients were excluded: Those with severe hypoalbuminemia (albumin <2.5 g/dL), diabetes mellitus, or hyperlipidemia (fasting serum triglyceride >250 mg/dL), those who were overweight (body mass index >30 kg/m^2^), had drug abuse or chronic alcoholism, liver disease (total bilirubin >2 mg/dL), renal disease (creatinine >2 mg/dL or needing hemodialysis), alternations of coagulation (thrombocytes < 150 × 10^3^/uL; international normalized ratio > 1.5; partial thromboplastin time > 40 sec), heart failure, life-threatening disease, those who were pregnant or lactating, those who received chemotherapy within 14 days before the start of the trial, had already accepted enteral nutrition, or participated in another clinical study with an investigational drug or an investigational medical device within a month prior to the start or during the study, and those who were hypersensitive to fish, egg, soy, or peanut protein. Before initiation of the study, a randomization sequence was computer-generated (GraphPad statistical software, GraphPad, USA) with two blocks of 60 patients each that was 1:1 allocated by an independent investigator (JFC). The patients were therefore assigned to either the study group or the control group and received the blinded study medication in identical appearance according to the randomization sequence by two investigators (CJM at KMUH and JMW at NTUH). All the investigators and staff, except for the independent investigator mentioned above, were kept blind to the assignment till the end of study. Based on the randomization sequence, emergency envelops were provided to the study centers to break blinding if reasonable suspicion of harm to the participants due to the investigational lipid emulsion was expressed. The emergency envelops were provided in three sets, one of which was installed at the ward another at the pharmacy of each respective hospital and the third one with the study coordinator of B. Braun Melsungen AG, Germany.

A total caloric supplement of 20–35 kcal/kg ideal body weight (BW)/day parenteral nutrition, including 1.2 g amino acid/kg BW/day, 3–4 g glucose/kg BW/day, 0.8-1.5 g lipid/kg BW/day and electrolyte, micro-element and vitamin according to the nutritional status of subjects and followed the standard procedure of the hospital, was infused continuously daily for the day before surgery and consecutive post-operative 7 days in an infusion time ranged between 18–24 hours. The doses of nutritional components were selected according to the European Society for Parenteral and Enteral Nutrition (ESPEN) guideline on parenteral nutrition for patients undergoing surgery. According to the assignment of the patients, the study group (MCT/LCT/n-3) received Lipoplus® 20% (B. Braun, Melsungen AG, Germany) and the control group (MCT/LCT) Lipofundin® MCT 20% (B. Braun, Melsungen AG, Germany), as part of the parenteral nutrition regimen provided to the subjects. The composition of each lipid emulsion is shown in Table 1. Energy expenditure is calculated according to Harris-Benedict Equation, and one or two bottles (usually one bottle) of lipid emulsion are administered to meet requirement of 0.8-1.5 g lipid/kg BW/day. The rest of calories needed are adjusted by glucose. Lipoplus® 20% contains 12.6-26.0 mg/ml of n-3 PUFAs and provided amount of 0.8-1.5 g lipid/kg BW/day is equivalent to 4–7.5 ml/kg BW/day. Approximately 80–140 mg/kg BW/day of n-3 PUFAs were provided in the study group. Enteral nutrition was restricted to clear liquid during 7 days postoperatively.

**Table 1:** **Composition of lipid emulsions**

	MCT/LCT/n-3 (Lipoplus®)	MCT/LCT (Lipofundin® MCT)
LCT (soybean oil), g/l	80	100
MCT, g/l	100	100
Fish oil, g/l	20	-
Egg phospholipids, g/l	12	12
Glycerol, g/l	25	25
Essential fatty acids		
LA (n-6 PUFA), g/l	38-46	48-58
ALA (n-3 PUFA), g/l	4.0-8.8	5-11
EPA and DHA (n-3 PUFA) , g/l	8.6-17.2	
n-6/n-3 fatty acid ratio	2.7:1	7-9:1
Total energy, kcal/l	1910	1910
pH	6.5-8.5	6.5-8.5
Osmolarity, mOsm/l	410	380

LCT: long-chain triglyceride; MCT: medium-chain triglyceride; LA: linoleic acid; PUFA: polyunsaturated fatty acid; ALA: α-linoleic acid; EPA: eicosapentaenoic acid; DHA: docosahexaenoic acid.

The primary objective was to evaluate the impact of n-3 PUFAs on the post-operative inflammatory response. Inflammatory markers used to assess inflammatory processes include IL-6, C-reactive protein (CRP), tumor necrosis factor-α (TNF-α) and procalcitonin (PCT). The respective levels were measured together with metabolic parameters on day -1, before and after surgery, and post-operative days 1, 3 and 7 as well as at follow up. Follow up was defined as 30 days after last treatment. Metabolic efficacy was measured by free fatty acids (FFA), triglycerides (TG), total cholesterol (TC), low-density lipoprotein (LDL), high-density lipoprotein (HDL), glucose and insulin. Metabolic efficacy was measured by the net changes (day 7 minus day -1).

Safety was assessed by hepatological variables including aspartate aminotransferase (AST), alanine aminotransferase (ALT), gamma-glutamyl transpeptidase (γGT), direct bilirubin and albumin, by hematological variables including international normalized ratio (INR), partial thromboplastin time (PTT), leukocyte-, platelet- and erythrocyte-count. All the hepatological and hematological safety parameters were measured on day -1, days 1, 3, and 7. Vital signs, fluid input, clinical course, concomitant medication, concomitant procedure, adverse events (AEs) and severe adverse events (SAEs) were recorded daily between day -1 and day 7 before administration of parenteral nutrition. Last visit of the patients was at discharge from hospital or 30 days after last treatment whatever occurred first. Clinical outcomes and AEs/SAEs were assessed until discharge of the patients from hospital. AEs/SAEs were coded using the MedDRA system (version 13.0, http://www.meddra.org) and summarized descriptively by system organ class.

To derive a sample size (equal in both study arms), it was required to specify the expected data regarding the primary end-point, the statistical significance level and the power. Based on published data about the influence of enteral nutrition regimen containing n-3 PUFAs on IL-6 release in Asian population, [16] we had conducted a 2-sided testing of independent means with the following values of IL-6. Treatment group (9 days after surgery – baseline) was 142 ± 118 pg/mL and control group (9 days after surgery – baseline) was 220 ± 124 pg/mL. The standard statistical significance of 5% and 80% power of rejecting null hypothesis when alternative hypothesis was corrected were chosen. The sample size calculation performs with software program nQuery version 5.0 results in 41 patients at least. Taking into a drop-out rate of 20%, the sample size is 50 patients per study arm i.e. a total 100 patients in the study.

Results are compared using Mann–Whitney U test or Kruskal-Wallis test for ordered categorical counts in case of non-paired data. In case of paired data the Wilcoxon rank-sum test or Friedman test is used. For the comparison of dichotomous variables the Fisher’s exact test is used. To variables of categorical character with more than two categories the Pearson chi-square test is applied. In case of paired categorical data the McNemar test is used. Means are compared using the 2-sample test and if appropriate using analysis of variance (ANOVA), where appropriate linear regression is performed. Yet, for all inferential analysis methods described above, the center effect is not considered when comparing one treatment to the other. Therefore, ANOVA incorporating center effect and Cochran-Mantel-Haenszel test stratified by center are applied to replace 2-sample t-test and Fisher’s exact test.

For efficacy analyses and part of the safety analyses (including of laboratory data and vital sign data), in order to consider the impact of baseline data on the endpoints, analysis of covariance (ANOVA) was applied when comparing a treatment mean to another, with their respective baseline as covariate. Baseline was defined as the data obtained before first administration of treatment before surgery. Endpoints were defined as net change of post-treatment from baseline. Statistical analyses were conducted with SPSS 18.0 (SPSS, Chicago, IL, USA).

## Results

**Figure 1:**
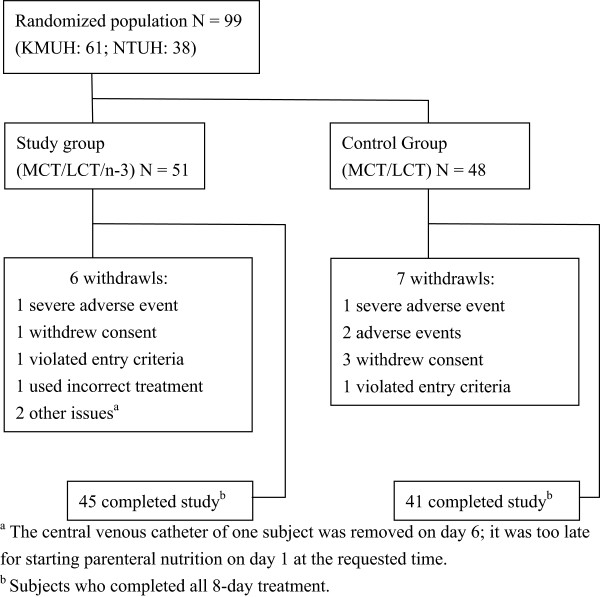
**Diagram of randomization of patients with gastric or colorectal cancer to receive an n-3 fatty acid enriched parenteral lipid emulsion or a control parenteral lipid emulsion (MCT/LCT).**

A total of 100 subjects were planned for enrollment in order to gain 82 evaluable subjects, with 41 in each treatment arm. Before the screening visit, a total of 128 patients diagnosed with gastric or colorectal cancer were each given a screening number by the study nurse. However, in site NTUH, 27 pre-screened patients neither signed the informed consent form nor had protocolized screening activities performed by investigators, and thus were excluded before the screening visit. The remaining 101 patients signed the informed consent form and had screening visits. Two subjects in site KMUH were screening failures. In the end, 99 patients who took at least one dose of study treatment had safety evaluations and comprised the safety population (51 in the study group, 48 in the control group). Figure 1 shows the disposition of subjects for the two treatment groups. A total of 8 subjects were withdrawn prior to post-operative day 1 and only received study medication on the day before surgery (i.e. day -1). Aside from the 8 subjects mentioned above, 5 subjects were also withdrawn from this trial and did not receive all scheduled lipid emulsion infusions (Table 2). The remaining 85 patients who received treatment medications from day -1 to day 7 and fulfilled all entry criteria were completers and where therefore included in the per protocol population.

**Table 2:** **List of subjects who were excluded from per protocol population**

Treatment	Reason for premature termination	Treatment duration
Study group (MCT/LCT/n-3)	Subject withdrew consent	1 day
	Subject withdrew from the study due to without pathological evidence of colon cancer	5 days
	Platelet at screening visit was 135 × 10^3^/uL, < 150 × 10^3^/uL	8 days
	Subject used incorrect treatment (used MCT/LCT)	1 day
	Patient experienced life-threatening SAE	7 days
	Central venous catheter was removed due to infection	6 days
	Too late for starting PN on day 1 at requested time	1 day
Control group (LCT/MCT)	AE: bilateral ankle pain	1 day
	Subject withdrew consent	1 day
	High fever, suspect central venous catheter infection	3 days
	Subject withdrew consent	1 day
	Acute bowel obstruction developed and patient underwent diversion colostomy and staged surgery	1 day
	Patient experienced life-threatening SAE	7 days
	Subject withdrew consent	1 day

SAE: severe adverse event; AE: adverse event.

The demographic characteristics of all subjects are summarized in Table 3. The groups were comparable with respect to demographic data with no difference in fluid input, concomitant medications, or concomitant procedures between the test groups. The distribution of tumor staging between test groups also seemed consistent.

**Table 3:** **Demographic data in our studied patients**

	Study group (N = 51)	Control Group (N = 48)	***P***
Sex			
Male/Female	29/22	27/21	0.960
Age (years)	61.55 ± 9.78	62.85 ± 10.12	0.530
Body weights (kg)	59.82 ± 11.04	61.55 ± 10.95	0.415
BMI (kg/m^2^)	23.45 ± 3.44	23.91 ± 3.79	0.501
Tumor staging – Gastric cancer			Total
N	14	12	26
UICC Stage I	4 (28.6%)	1 (8.3%)	5 (19.2%)
Stage II	3 (21.4%)	4 (33.3%)	7 (26.9%)
Stage III	4 (28.6%)	2 (16.7%)	6 (23.1%)
Stage IV	3 (21.4%)	5 (41.7%)	8 (30.8%)
Tumor staging – Colorectal cancer			
N	36	37	73
UICC Stage I	3 (8.3%)	3 (8.1%)	6 (8.2%)
Stage II	15 (41.7%)	12 (32.4%)	27 (37.0%)
Stage III	14 (38.9%)	15 (40.5%)	29 (39.7%)
Stage IV	4 (11.1%)	7 (18.9%)	11 (15.1%)

UICC: union of international cancer control.

All laboratory parameters were comparable in treatment groups at baseline, and similar trends between treatment groups were observed throughout the study period (Table 4). However, statistically significant differences between groups were detected in the net changes of FFA, TG and HDL from day -1 to post-operative visits. Patients in study group (i.e. MCT/LCT/n-3) showed a significantly stronger reduction in net change of FFA when compared with the control group (i.e. MCT/LCT) from day -1 to day 7 (-0.201 mmol/L vs. -0.026 mmol/L, p = 0.013). Moreover, patients from both groups had increments in TG levels after surgery, but the increment from day -1 to day 7 was less in the study group as compared with the control group (20.98 mg/dl vs. 66.69 mg/dl, p = 0.0006). Lastly, both groups exhibited statistically significant differences in net change of HDL from day -1 to day 3 (-0.85 mg/dl vs. -6.11 mg/dl, p = 0.0097) and from day -1 to day 7 (-12.58 mg/dl vs. -17.36 mg/dl, p = 0.0099), where HDL level reduction was less in the study group. Despite the differences, the aforementioned parameters were all within normal range.

**Table 4:** **Various laboratory data before and after operation between the two studied groups**

	Study group (N = 44)	Control group (N = 41)	Difference ^1,2^ [95% C.I.]	***P***
FFA (mmol/L)				
Day -1	0.60 ± 0.35	0.65 ± 0.41	-0.044 [-0.196; 0.108]	0.566
Day 7 – Day -1	-0.20 ± 0.38	-0.03 ± 0.62	-0.195 [-0.347; -0.042]	0.013
TG (mg/dL)				
Day -1	97.45 ± 41.12	95.06 ± 38.72	2.263 [-13.714; 18.239]	0.779
Day 7 – Day -1	20.98 ± 44.85	66.69 ± 68.85	-44.109 [-68.583; -19.634]	<0.001
Cholesterol (mg/mL)				
Day -1	179.22 ± 34.27	177.77 ± 39.04	1.188 [-13.225; 15.600]	0.870
Day 7 – Day -1	-42.61 ± 32.00	-40.71 ± 34.86	-2.001 [-13.549; 9.546]	0.731
LDL (mg/mL)				
Day -1	110.99 ± 23.93	109.40 ± 36.00	1.660 [-10.504; 13.823]	0.787
Day 7 – Day -1	-28.70 ± 25.76	-27.41 ± 30.50	-1.350 [-11.281; 8.581]	0.788
HDL (mg/mL)				
Day -1	37.85 ± 12.62	40.57 ± 14.87	-2.889 [-8.031; 2.254]	0.268
Day 7 – Day -1	-12.58 ± 8.70	-17.36 ± 10.01	3.226 [0.796; 5.656]	0.010
Insulin (mU/L)				
Day -1	14.14 ± 14.96	21.80 ± 26.50	-7.547 [-16.107; 1.013]	0.083
Day 7 – Day -1	22.44 ± 24.68	9.80 ± 29.44	8.080 [-1.870; 18.029]	0.110
Glucose (mg/dL)				
Day -1	133.31 ± 57.80	128.92 ± 52.68	4.840 [-16.791; 26.472]	0.658
Day 7 – Day -1	20.00 ± 47.80	29.91 ± 76.32	-6.656 [-30.295; 16.983]	0.577
ALT (U/L)				
Day -1	18.65 ± 7.65	18.73 ± 12.16	-0.010 [-3.976; 3.956]	0.996
Day 7 – Day -1	25.35 ± 33.30	38.48 ± 43.24	-11.141 [-26.835; 4.552]	0.162
AST (U/L)				
Day -1	23.77 ± 11.75	23.46 ± 11.80	0.325 [-4.394; 5.044]	0.892
Day 7 – Day -1	18.22 ± 34.51	24.62 ± 34.26	-4.523 [-18.384; 9.337]	0.518
γGT (U/L)				
Day -1	21.88 ± 18.64	28.10 ± 47.67	-5.943 [-20.257; 8.371]	0.412
Day 7 – Day -1	78.27 ± 71.93	118.24 ± 112.41	-36.052 [-73.770; 1.667]	0.061
Albumin (g/dL)				
Day -1	3.98 ± 0.47	4.01 ± 0.46	-0.035 [-0.194; 0.124]	0.662
Day 7 – Day -1	-0.42 ± 0.38	-0.51 ± 0.39	0.099 [-0.038; 0.236]	0.153
Bilirubin (mg/dL)				
Day -1	0.15 ± 0.10	0.15 ± 0.08	0.002 [-0.034; 0.037]	0.929
Day 7 – Day -1	0.04 ± 0.26	0.09 ± 0.33	-0.052 [-0.171; 0.067]	0.389
Leukocyte (10^3^/μL)				
Day -1	5.91 ± 2.52	8.00 ± 12.85	-2.121 [-5.764; 1.522]	0.251
Day 7 – Day -1	28.41 ± 174.51	0.67 ± 14.01	23.872 [-31.335; 79.079]	0.392

FFA: free fatty acid; TG: triglyceride; LDL: low density lipoprotein; HDL: high density lipoprotein; ALT: alanine aminotransferase; AST: aspartate aminotransferase; γGT; gamma-glutamyl transpeptidase.

^1^Difference = [study group] - [control group].

^2^ANOVA incorporating center effect and their respective baseline as covariate; Difference and 95% C.I. was based on the LS-mean.

**Figure 2:**
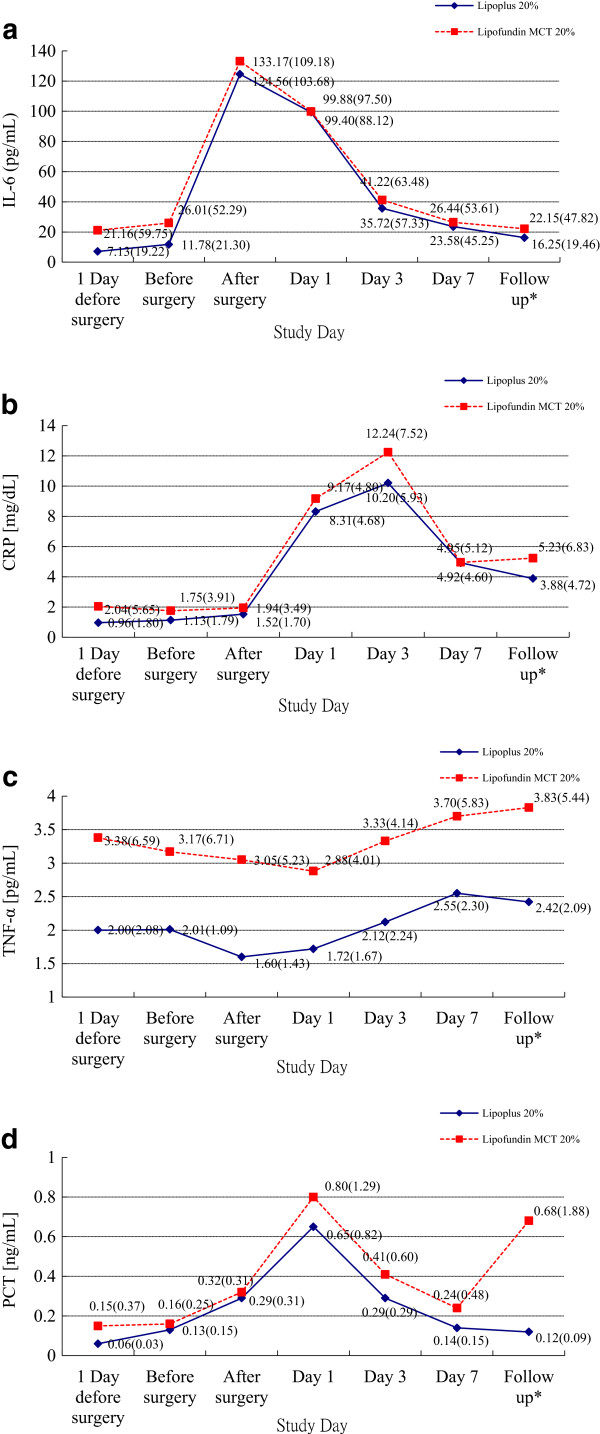
**Distribution of mean (SD) during the study period. (a)** IL-6, **(b)** CRP, **(c)** TNF-α, **(d)** PCT. *Follow up was defined as 30 days after last treatment.

Pro-inflammatory factors (IL-6, CRP, TNF-α, and PCT) were comparable in the two groups, but no prominent differences were observed (Table 5). Despite a higher level of pro-inflammatory factors in the control group at each visit time, the two treatment groups did not show any statistically significant difference. The two groups demonstrated consistent trends in pro-inflammatory factors levels during the study period. The IL-6 level immediately reached the peak after surgery and the CRP and the PCT levels reached the peak on day 3 and day 1 respectively. All three parameters gradually decreased afterwards and there was no statistical difference on each day it was measured between test groups. The level of TNF-α didn’t vary obviously and had no significant difference between two groups, either (Figure 2).

**Table 5:** **Various inflammatory factors before and after operation between the two studied groups**

	Study group (N = 44)	control group (N = 41)	Difference ^1,2^ [95% C.I.]	***P***
IL-6 (pg/mL)				
Day -1	7.13 ± 19.22	21.16 ± 59.75	-13.724 [-32.496; 5.048]	0.150
Before surgery	11.78 ± 21.30	26.01 ± 52.29	-13.793 [-30.417; 2.824]	0.103
After surgery	126.56 ± 103.68	133.17 ± 109.18	-8.867 [-55.025; 37.291]	0.703
Day 1	99.40 ± 88.12	99.88 ± 97.50	-0.958 [-41.005; 39.089]	0.962
Day 3	35.72 ± 57.33	41.22 ± 63.48	-5.808 [-31.878; 20.262]	0.659
Day 7	23.58 ± 45.25	26.44 ± 53.61	-2.741 [-24.202; 18.719]	0.800
Follow up^3^	16.25 ± 19.46	22.15 ± 47.82	-5.810 [-21.446; 9.826]	0.462
Day 7 – Day -1	16.45 ± 28.31	5.27 ± 53.00	6.648 [-10.876; 24.172]	0.453
CRP (mg/dL)				
Day -1	0.96 ± 1.80	2.04 ± 5.65	-1.069 [-2.857; 0.720]	0.238
Before surgery	1.13 ± 1.79	1.75 ± 3.91	-0.615 [-1.919; 0.688]	0.350
After surgery	1.52 ± 1.70	1.94 ± 3.49	-0.405 [-1.576; 0.765]	0.493
Day 1	8.31 ± 4.68	9.17 ± 4.80	-0.837 [-2.877; 1.202]	0.416
Day 3	10.20 ± 5.93	12.24 ± 7.52	-2.043 [-4.792; 0.885]	0.169
Day 7	4.92 ± 4.60	4.95 ± 5.12	-0.040 [-2.152; 2.072]	0.970
Follow up^3^	3.88 ± 4.72	5.23 ± 6.83	-1.304 [-3.804; 1.196]	0.303
Day 7 – Day -1	3.95 ± 4.89	3.01 ± 7.11	-0.004 [-2.155; 2.148]	0.997
TNF-α (pg/mL)				
Day -1	2.00 ± 2.07	3.38 ± 6.59	-1.395 [-3.474; 0.685]	0.186
Before surgery	2.01 ± 1.09	3.17 ± 6.71	-1.176 [-3.224; 0.872]	0.257
After surgery	1.60 ± 1.43	3.05 ± 5.23	-1.475 [-3.096; 0.147]	0.074
Day 1	1.72 ± 1.67	2.88 ± 4.01	-1.174 [-2.485; 0.137]	0.079
Day 3	2.12 ± 2.24	3.33 ± 4.14	-1.237 [-2.653; 0.180]	0.086
Day 7	2.55 ± 2.30	3.70 ± 5.83	-1.169 [-3.061; 0.724]	0.223
Follow up^3^	2.42 ± 2.09	3.83 ± 5.44	-1.424 [-3.182; 0.334]	0.111
Day 7 – Day -1	0.55 ± 1.38	0.38 ± 1.63	0.002 [-0.609; 0.612]	0.995
PCT (ng/mL)				
Day -1	0.06 ± 0.03	0.15 ± 0.37	-0.085 [-0.197; 0.027]	0.133
Before surgery	0.13 ± 0.15	0.16 ± 0.25	-0.029 [-0.118; 0.060]	0.518
After surgery	0.29 ± 0.31	0.32 ± 0.31	-0.029 [-0.163; 0.105]	0.671
Day 1	0.65 ± 0.82	0.80 ± 1.29	-0.148 [-0.612; 0.316]	0.528
Day 3	0.29 ± 0.29	0.41 ± 0.60	-0.125 [-0.325; 0.075]	0.216
Day 7	0.14 ± 0.15	0.24 ± 0.48	-0.097 [-0.249; 0.056]	0.211
Follow up^3^	0.12 ± 0.09	0.68 ± 1.88	-0.557 [-1.120; 0.006]	0.053
Day 7 – Day -1	0.08 ± 0.15	0.09 ± 0.62	-0.101 [-0.256; 0.055]	0.202

CRP: C-reactive protein; TNF-α: tumor necrosis factor-α; PCT: procalcitonin.

^1^Difference = [study group] - [control group].

^2^ANOVA incorporating center effect and their respective baseline as covariate; Difference and 95% C.I. was based on the LS-mean.

^3^Follow up was defined as 30 days after last treatment.

The extent of exposure, in terms of days of 99 patients exposed to lipid emulsion was 7.49 days for the study group, and 7.17 days for the control group. Moreover, both groups demonstrated median extent of exposing as 8 days. There was no significant difference in the extent of exposure between treatment groups (p = 0.399).

A total of 6 patients experienced pre-treatment AEs, having 2 (3.9%) vs. 4 (8.3%) of subjects in the study vs. control group, respectively. No significant difference was observed (p = 0.371). A total of 314 incidents of treatment-emergent AEs had occurred in 88 (88.9%) subjects, with 47 (92.2%) and 41 (85.4%) subjects in study and control group, respectively (p = 0.243). The most reported AEs were injuries; poisoning, procedural complications and general disorders and administration site conditions were the second most frequently occurring AEs (Table 6). There were no particular treatment group differences concerning the category and incidence of treatment-emergent AEs.

**Table 6:** **Number (%) of treatment-emergent AEs with incidence > 5% by body system – safety population**

System organ class		Study group (N = 51)	Control group (N = 48)	Total (N = 99)
Gastrointestinal disorders	Abdominal distension	3 (5.9%)	4 (8.3%)	7 (7.1%)
	Nausea	4 (7.8%)	1 (2.1%)	5 (5.1%)
	Vomiting	4 (7.8%)	1 (2.1%)	5 (5.1%)
General disorders and administration site conditions	Pyrexia	24 (47.1%)	28 (58.3%)	52 (52.5%)
Infections and infestations	Wound infection	3 (5.9%)	1 (2.1%)	4 (4.0%)
Injury, poisoning, and procedural complications	Wound complication	40 (78.4%)	33 (68.8%)	73 (73.7%)
Investigations	ALT increased	6 (11.8%)	6 (12.5%)	12 (12.1%)
	AST increased	5 (9.8%)	6 (12.5%)	11 (11.1%)
	CRP increased	5 (9.8%)	9 (18.8%)	14 (14.1%)
	γGT increased	10 (19.6%)	11 (22.9%)	21 (21.2%)
	White blood count increased	2 (3.9%)	7 (14.6%)	9 (9.1%)
Metabolism and nutrition disorders	Hypoalbuminemia	5 (9.8%)	2 (4.2%)	7 (7.1)

ALT: alanine aminotranferase; AST: aspartate aminotranferase; CRP: C-reactive protein; γGT: gamma-glutamyltransferase.

Approximately 80% of AEs were related as mild in intensity and nearly one-fifth of AEs were moderate. Only 5 AEs were severe in intensity, including 2 SAEs. One of the SAEs occurred in a subject in the study group with wound complication, septic shock and blood pressure decreased. Another was a subject reported with septic shock in the control group. Both SAEs were observed on day 7 and caused the withdrawal of both subjects from the study. AST was the only AE which was possibly related to trial medication. This AE was reported as mild in intensity and no action was taken regarding this episode. No death was encountered in this study.

## Discussion

Patients considered for major surgery for gastric and colorectal cancer are usually malnourished before surgery, and very likely to undergo a worsening of this status, because of surgery induced stress metabolism leading to an increase in morbidity and mortality. Nonetheless, nutritional intervention may substantially alter the nutritional status as well as the immune function with potential impact on the post-operative course. Lipid emulsions containing n-3 PUFAs, namely eicosapentaenoic acid (EPA) and docosahexanoic acid (DHA), are introduced into the international market of PN. Next to provision of energy, studies have demonstrated that these n-3 PUFAs may regulate pro- and anti-inflammatory cytokine gene expression and compete with AA for the enzymes involved in eicosanoid metabolism and thus might represent a promising therapy in conditions characterized by inappropriate pro-inflammatory activity [9].

IL-6, CRP and TNF-α are common and sensitive but non-specific markers of inflammatory processes and increased concentrations are measured after trauma and in sepsis. Thus, they are chosen to evaluate immune modulating effects of n-3 PUFAs. To complement the inflammatory assessment, PCT as an early and highly sensitive marker of inflammation of bacteriological origin is included into the analysis. PCT is also considered to serve as decision marker for any needed antibiotic treatment.

In fact, there is clinical evidence showing anti-inflammatory and immunomodulatory effects of n-3 PUFAs on rheumatoid arthritis [17–19], lupus [20, 21], and inflammatory bowel disease [22–24], which are hyperinflammatory autoimmune diseases. Several studies have demonstrated that n-3 fatty acids can modulate the immune response as determined by an improved eicosanoids profile [15, 16, 25, 26]. In surgical patients, the earlier study of Wachtler et al. could show significant attenuating effects of n-3 fatty acids on IL-6 levels in patients undergoing elective major surgery [15]. Also in the study by Chen et al. [16], on which the case number calculation for our current trial was performed, lower postoperative IL-6 levels of patients undergoing major gastrointestinal surgery were reported in the n-3 fatty acid group.

The levels of IL-6, TNF-α, CRP, and PCT in study group are all significantly lower than control group at each visit time and therefore, n-3 PUFAs have a trend to reduce pro-inflammatory factors. However, consistent with our previously published study, [11] our results show that n-3 PUFAs containing lipid emulsion as part of PN does not significantly influence levels of pro-inflammatory factors, including IL-6, TNF-α, CRP, and PCT, or clinical outcomes in patients, undergoing elective surgery. Noteworthy, in the study by Chen et al., administration of enteral immunonutrition in patients with gastric carcinoma undergoing major surgery, the IL-6 values on the day of surgery, directly after surgery, are reported above/on average 270–299 pg/ml. On post-operative day 1 the IL-6 values are even as high as 584 pg/ml (mean). After 9 days, the values of IL-6 are still high (mean 411 pg/ml in the immunonutrition group, mean 519 pg/ml in the control group). Thus, the inflammatory response as judged by the IL-6 values seem to completely different in the study by Chen et al. that IL-6 being 5 times higher than in the current study. This may be explained by the use of minimal invasive surgery in our study compared to major surgical interventions in the previous one. Again, PCT, as an early highly sensitive marker of inflammation of bacterial origin, elevated slightly in both groups in conjunction with other inflammatory parameters also indicates that the inflammatory response in our patients is mild. As we expected a stronger inflammatory response, and calculated the case number accordingly, the effect of n-3 PUFAs on cytokine expression might be masked and the resulting measurable effects on the inflammatory response are only mild.

Surgical trauma induces a catabolic response with hypercatabolism and impaired lipid tolerance [27, 28]. Hepatic triglyceride synthesis is related to the availability of carbohydrate and fatty acid substrates [29]. As a result, patients receiving postoperative PN may present hyperlipidemia. In this study, the postoperative levels of TG are elevated in both groups; however, the increase is moderate and remains within normal ranges. The clearance of FFA and TG in the study group is better than in the control group, and elimination is adequate without hypertriglyceridemia. Furthermore, decrease of HDL is also less in the study group compared to the control. However, metabolism of lipoprotein is complex and many factors may influence the residence time. Although results from Annuzzi et al. [30] demonstrates that administration of n-3 PUFAs is associated with favorable lipoprotein profile, it is a surveillance of lipoprotein and dietary n-3 PUFAs but it is difficult to conclude that short time of n-3 PUFAs infusion is also associated with a better cholesterol profile.

Except for FFA, TG and HDL, laboratory parameters regarding hepatological safety, hematological safety, lipid and glucose metabolism as well as AEs are comparable in both treatment groups. There is a trend in increased level of AST, ALT and γGT as well as a trend in increased level of corresponding FFA and TG. Increased FFA and TG in blood, i.e. hyperlipidemia, may theoretically increase fat storage in the liver, cause inflammation in the liver and eventually result non-alcoholic fatty liver disease (NAFLD). It is an observation within a short period window, though this may explain the association of lipid profile and elevated liver enzymes. The only AE which is possibly treatment-related is a mild AST elevation. It is well known that TPN may alter liver function tests after days of administration, and accordingly elevated AST and ALT without a change in serum total bilirubin levels. The mechanisms underlying such abnormalities are not fully understood, although they may partially result from the lack of gastrointestinal stimulation by enteral nutrition. Two subjects, one in each treatment group, were documented with life-threatening SAE of septic shock, and the study treatment was interfered regarding these episodes. However, both SAEs were unrelated to study medication and no death was recorded.

This study has limitations. First of all, the patients selected are those who received elective surgery for gastrointestinal cancers, instead of critically ill patients with surgery for inflammatory or infectious diseases, such as inflammatory bowel disease, peritonitis or necrotizing pancreatitis and so on. The expected immunomodulatory effects may be masked. Secondly, it is a treatment in a short time, and only relative changes of several biomarkers can be observed. Finally, our current study might not have been sufficiently powered to draw significant conclusions, and the detection of significant effects in surgical patients under contemporary conditions (i.e. using minimal invasive surgery) might require further and larger clinical trials.

## Conclusions

Both lipid emulsions exerted a comparable effect on the efficacy parameters chosen and n-3 PUFAs had limited immunomodulation in normal subjects undergoing elective surgery for gastric and colorectal cancers. Despite being a small-population study with some limitations mentioned above, n-3 PUFAs may support better lipid elimination; less development of hyperlipidemia and n-3 PUFAs containing lipid emulsion is safe and well tolerated as a standard lipid emulsion in the immediate postoperative period. The expected immunomodulation is unfortunately failed to be observed.

## Author’s information

Cheng-Jen Ma is a surgeon in the division of gastrointestinal and general surgery and also a member of nutrition support team at Kaohsiung Medical University Hospital, Taiwan.

## Abbreviations

LCT: Long-chain triglyceride
MCT: Medium-chain triglyceride
PUFA: Poly-unsaturated fatty acid
IVFE: Intravenous fat emulsion
TPN: Total parenteral nutrition
FFA: Free fatty acid
TG: Triglyceride
ARDS: Acute respiratory distress syndrome
BW: Body weight
ESPEN: European society for parenteral and enteral nutrition
PCT: Procalcitonin
TC: Total cholesterol
ANOVA: Analysis of variance
AE: Adverse event
SAE: Sever adverse event
EPA: Eicosapentaenoic acid
DHA: Docosahexanoic acid
NAFLD: Non-alcoholic fatty liver disease.
